# Transactions of the American Medical Association

**Published:** 1852-02

**Authors:** 


### TRANSACTIONS OF THR AMERICAN MEDICAL ASSOCIATION.

We have read the following announcement, in the *Medical News*, with extreme regret. The destruction of so large a portion of this national work will be felt as a personal loss by many members of the profession who have not yet supplied themselves with copies of it.

"We stop the press to announce the unpleasant intelligence that, since the preceding notice was in type, a very large and disastrous fire has occurred in this city, in which two-thirds of the edition of the fourth volume of the Transactions was consumed, with nearly all the previous volumes remaining on hand. Fortunately, the fourth volume had been distributed to nearly all the members of the Association who had paid their assessment, and copies for the others who had done so are at the store of Messrs. Blanchard and Lea, and are safe. Such members of the Association, as neglected to take advantage of the highly favorable terms upon which the Transactions were offered, have now, unfortunately lost the opportunity. The few remaining copies will be reserved a short time for members, but probably at an advanced price, and, if not shortly claimed, wtll be sold to other applicants."

